# Sirtuin Expression in Age-Associated Hepatic Response to Burn Trauma: Translational and Clinical Insights From a Murine Model

**DOI:** 10.7759/cureus.82663

**Published:** 2025-04-20

**Authors:** Kenneth Meza Monge, Andrea C Qualman, Akshay Pratap, Elizabeth J Kovacs, Juan-Pablo Idrovo

**Affiliations:** 1 Department of Surgery, Division of Gastrointestinal, Trauma, and Endocrine Surgery, University of Colorado Anschutz Medical Campus, Aurora, USA

**Keywords:** aging, apoptosis, burn injury, gene expression, hepatic damage, inflammation, liver injury, oxidative stress, sirtuins, trauma

## Abstract

Background

Burn injuries can lead to substantial liver damage, and this response appears to worsen with age. Although clinical patterns suggest that older individuals are more susceptible to poor outcomes, the biological mechanisms contributing to this increased vulnerability are poorly understood. Sirtuins, a family of nicotinamide adenine dinucleotide (NAD^+^)-dependent enzymes involved in cellular stress regulation, metabolism, and aging, may play a key role in modulating the hepatic response to burn injury. This study explores the potential mechanistic involvement of sirtuins in age-related liver damage following thermal injury.

Methods

Female C57BL/6 mice (young: four months; aged: 20-22 months) underwent sham or 15% total body surface area scald burn. Liver tissue was collected at 24- and 48-hours post-injury for quantitative polymerase chain reaction (qPCR) analysis of all seven sirtuin family members.

Results

Burn injury significantly altered hepatic sirtuin expression in an age- and time-dependent manner. Inflammatory regulators, *Sirt1* and *Sirt2*, showed immediate downregulation in young mice with partial recovery at 48 hours, while aged mice exhibited delayed, more profound, and persistent suppression. In contrast, mitochondrial sirtuins (*Sirt3-5*) were downregulated in both age groups, and only young burned mice showed recovery of *Sirt3* and *Sirt4* expression at 48 hours. The most pronounced age-dependent difference occurred with *Sirt4*. At this time point, the expression of *Sirt4* was 71% higher in young burned mice compared to aged injured counterparts (p < 0.05). Genome stability regulating *Sirt6* and *Sirt7* exhibited age-specific responses, with *Sirt6* remaining stable in young injured mice, while *Sirt7* was lower in aged mice at 48 hours (p < 0.05).

Conclusion

This study reveals for the first time that burn injury triggers age-dependent alterations in the pattern of hepatic sirtuin expression, with delayed and/or more severe and persistent suppression across all sirtuin family members. These findings provide new mechanistic insights into the dysregulation of critical cellular homeostatic mechanisms in aged livers following burn injury and identify sirtuins as potential therapeutic targets for mitigating age-associated hepatic damage in elderly burn patients.

## Introduction

Severe burn injuries represent a significant clinical challenge, particularly in the elderly population, who experience markedly higher rates of complications, prolonged hospitalization, and two- to three-fold increased mortality compared to younger patients [1]. This age-associated vulnerability has been documented across multiple burn centers, with elderly patients (>65 years) exhibiting mortality rates of 45-50% compared to 10-15% in younger adults with comparable burn injuries [1]. As stated by Jeschke, the liver, as a central metabolic hub regulating acute-phase proteins, cytokine production, and immune modulation, plays a crucial role in systemic response to any type of trauma, including burns [2]. However, age-related impairments in hepatic resilience can exacerbate systemic inflammation and organ dysfunction, predisposing elderly burn patients to adverse outcomes [3,4].

Our laboratory has previously demonstrated that the liver exhibits significant morphological and functional changes following cutaneous burns, with these alterations markedly exacerbated in aged animals [5,6]. Specifically, we observed elevated liver injury markers (alanine aminotransferase (ALT), aspartate aminotransferase (AST)), increased hepatic lipid deposits, heightened oxidative stress (measured by malondialdehyde (MDA) levels), and accentuated histological evidence of hepatic damage in aged mice compared to young counterparts after burn injury [5]. These findings highlight the increased vulnerability of the aged liver to acute stressors, but the molecular mechanisms driving this age-dependent susceptibility remain incompletely understood.

The molecular basis for heightened hepatic vulnerability in the aged appears to involve multiple interconnected pathways. Research from 2018 to 2022 demonstrates that burn-induced hepatic damage is driven not only by cytokine dysregulation but also by alterations in cellular stress response mechanisms, including sirtuin-mediated pathways [7,8]. Sirtuins are a family of 7 nicotinamide adenine dinucleotide (NAD^+^)-dependent deacetylases, named from *Sirt1* to *Sirt7*, that function as master regulators of cellular homeostasis. Based on their subcellular localization and function, sirtuins can be categorized into three groups: inflammatory regulators (*Sirt1* and *Sirt2*), mitochondrial function and oxidative stress regulators (*Sirt3*, *4*, and *5*), and genome stability and DNA repair modulators (*Sirt6* and *7*) [9]. These evolutionarily conserved proteins serve as critical sensors of cellular stress and metabolic state, orchestrating adaptive responses to maintain homeostasis under challenging conditions.

In our recent report on the transcriptomic and metabolomic analysis of liver tissue following burn injury, we identified significant alterations in pathways associated with sirtuin activity, including mitochondrial function, oxidative stress responses, and lipid metabolism [10]. The dysregulation of sirtuin expression has been implicated in aging-related hepatic vulnerability, including diminished capacity to respond to oxidative stress, impaired metabolic flexibility, and compromised cellular repair mechanisms [11]. However, the specific role of sirtuins in burn-induced liver injury, particularly in the context of aging, remains unexplored.

Comprehensive genetic analyses are essential to understanding how the liver responds to burn injuries differ between young and aged individuals. The present study aims to characterize hepatic sirtuin expression patterns following burn injury using a clinically validated murine burn model that closely replicates human burn physiology [12-14]. Given the crucial role of sirtuins in modulating inflammation, oxidative stress, and metabolic adaptation, identifying age-related changes in their expression may provide insight into the mechanisms driving burn-induced hepatic dysfunction. Ultimately, these findings could lead to targeted therapeutic strategies such as sirtuin modulators or epigenetic therapies, which are currently under investigation in cancer and autoimmune diseases, but may be repurposed and adapted to mitigate liver dysfunction after burns to improve care and clinical outcomes in elderly patients.

## Materials and methods

Mice

Young (four months old, equivalent to 20-25 human years) and aged (20-22 months old, comparable to 65-70 human years) female C57BL/6 mice were obtained from the Jackson Laboratory (Bar Harbor, ME, USA) and the National Institute on Aging (NIA) Colony at Charles River Laboratories (Wilmington, MA, USA). This age equivalence is based on established lifespan correlations between mice and humans as described by Flurkey et al. [15]. Prior to the study, all mice were housed at the University of Colorado Anschutz Medical Campus Vivarium for a minimum of two weeks to allow for acclimatization. Burn procedures were conducted between 9:00 AM and 11:00 AM to mitigate circadian variations in inflammatory responses and stress hormone levels, which can significantly impact experimental outcomes [16]. Measures were taken to minimize discomfort and distress, with continuous monitoring post-burn to assess signs of pain. Euthanasia was carried out following Institutional Animal Care and Use Committee (IACUC) guidelines (protocol no. 00001163), using a gradual CO_2_ exposure method followed by cervical dislocation as previously described [17]. All procedures were conducted in compliance with the ARRIVE (Animal Research: Reporting of In Vivo Experiments) guidelines for reporting animal research.

Clinical murine burn injury model

**Figure 1:**
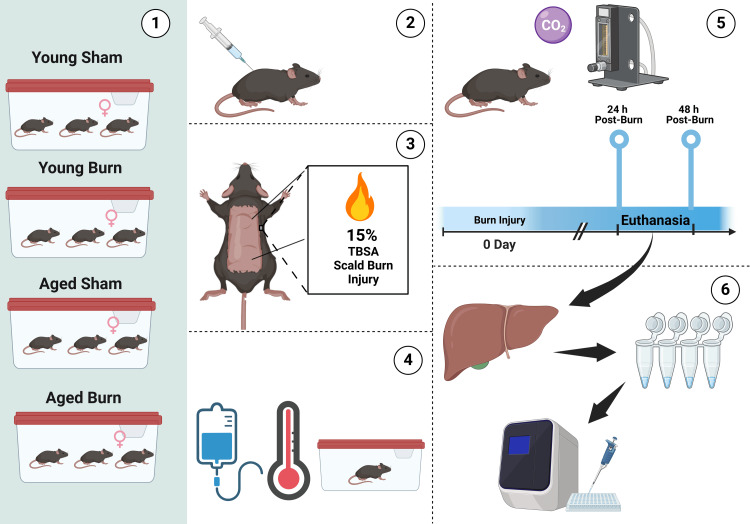
Schematic representation of the experimental design and workflow for assessing hepatic sirtuin expression following burn injury in young and aged mice (1) Experimental groups: female C57BL/6 mice were divided into four groups: young sham, young burn, aged sham, and aged burn. (2) Anesthesia and preparation: mice had the dorsum shaved and were anesthetized before undergoing procedures. (3) Burn injury induction: a full-thickness 15% total body surface area (TBSA) scald burn injury was administered by immersing the dorsal skin in 95°C water for 10 seconds, while sham controls were exposed to room-temperature water. (4) Post-burn care: mice received fluid resuscitation and analgesia, were housed under controlled conditions, and monitored for distress. (5) Euthanasia and tissue collection: groups of mice were euthanized at 24 or 48 hours post-burn via CO_2_ inhalation, and liver tissues were harvested. (6) Sample processing and analysis: liver samples were snap-frozen for RNA isolation and quantitative polymerase chain reaction (PCR). This image was created by the author (Kenneth Meza Monge) using BioRender (https://BioRender.com/y26b685).

Animals were randomly distributed into four experimental groups: young sham, young burn, aged sham, and aged burn, with eight mice initially allocated to each group at each time point. The final analysis included six to eight mice per group due to technical issues with RNA extraction in a small number of samples. Sample size was determined based on previous studies with similar endpoints that demonstrated statistical significance with n=6-8 per group [5,13]. Mice were anesthetized using ketamine (55.5 mg/kg) and xylazine (2.6 mg/kg) before undergoing a full-thickness, 15% total body surface area (TBSA) scald burn injury. The dorsum was shaved, and mice were immersed in 95°C water for 10 seconds, a technique that ensures consistent burn depth and severity as previously validated and described [12,13,18]. Control (sham) mice underwent identical handling and were exposed to room-temperature water (25°C). To mimic clinical post-burn care, all mice received analgesia (buprenorphine extended-release, 0.13 mg/kg) and fluid resuscitation (1 mL subcutaneous normal saline) and were kept normothermic in their cages using warm pads. Mice were monitored for distress, and euthanasia was performed at either 24 or 48 hours post-burn, timepoints selected based on previous studies demonstrating significant hepatic changes within this critical period (Figure 1) [5].

RNA isolation and quantitative gene expression analysis

Liver tissue samples were homogenized in TRIzol reagent (no. 15596018, Life Technologies, Carlsbad, CA, USA), and total RNA was extracted using chloroform (no. C2432, Sigma-Aldrich, St. Louis, MO, USA) followed by isopropanol precipitation (no. BP2618-1, Thermo Fisher Scientific, Waltham, MA, USA). RNA quality and concentration were assessed using spectrophotometry, with 260/280 ratios consistently >1.8 across all samples included in the analysis. Complementary DNA (cDNA) was synthesized using the iScript cDNA Synthesis Kit (no. 1708891, Bio-Rad, Hercules, CA, USA) according to the manufacturer's instructions, with 1 μg of total RNA per reaction. Quantitative reverse transcription polymerase chain reaction (RT-qPCR) was performed using Universal SYBR Green Supermix (no.1725124, Bio-Rad). Standard desalted primers were purchased from Sigma-Aldrich for *Sirt1* to *Sirt7* (Table 1). Glyceraldehyde 3-phosphate dehydrogenase (GAPDH) was used as an endogenous control (no. 4352339E, Fisher Scientific, Waltham, MA, USA), which has been validated in multiple burn injury studies as maintaining stable expression across experimental conditions [17,18]. Each sample was run in triplicate to minimize technical variation, and samples with a coefficient of variation >5% between technical replicates were re-run. PCR cycling conditions were initial denaturation at 95°C for three minutes, followed by 40 cycles of denaturation at 95°C for 15 seconds and annealing/extension at 60°C for 60 seconds. Melt curve analysis was performed to confirm amplification specificity. Plates were run and analyzed with QuantStudio 3 RealTime PCR System (Applied Biosystems, Thermo Fisher Scientific, Waltham, MA, USA) using the comparative threshold cycle (ΔΔCt) algorithm [19]. qPCR product specificity was confirmed by single melt peaks and consistent amplification plots, as shown in Appendices A-C.

**Table 1:** Primer sequences used for quantitative PCR analysis of sirtuin gene expression PCR: polymerase chain reaction; GAPDH: glyceraldehyde 3-phosphate dehydrogenase

Primer	Forward sequence (5'-3')	Reverse sequence (5'-3')
Sirt1	GGAGCAGATTAGTAAGCGGCTTG	GTTACTGCCACAGGAACTAGAGG
Sirt2	CGAAGGAGTGACACGCTACATG	GGTGGTACTTCTCCAGGTTTGC
Sirt3	GCTACATGCACGGTCTGTCGAA	CAATGTCGGGTTTCACAACGCC
Sirt4	GTGGATGCTTTGCACACCAAGG	GGTTCAGGACTTGGAAACGCTC
Sirt5	ATCGCAAGGCTGGCACCAAGAA	CTAAAGCTGGGCAGATCGGACT
Sirt6	CAGTACGTCAGAGACACGGTTG	GTCCAGAATGGTGTCTCTCAGC
Sirt7	CTGGAGATTCCTGTCTACAACCG	AGTGACTTCCTACTGTGGCTGC
GAPDH	CATCACTGCCACCCAGAAGACTG	ATGCCAGTGAGCTTCCCGTTCAG

Statistical analysis

Data are expressed as mean ± standard error of the mean (SEM), with n = 6-8 per group. Statistical analyses were performed using GraphPad Prism 10.2.3 (GraphPad Software, San Diego, CA, USA). For comparisons across multiple groups, one-way analysis of variance (ANOVA) with Tukey's post hoc test was used to identify significant differences between specific groups. Normality of data distribution was assessed using the Shapiro-Wilk test prior to parametric analysis, with all data sets confirming to normal distribution. Homogeneity of variance was confirmed using Levene's test. A p-value of <0.05 was considered statistically significant. The findings presented were derived from three independent murine experiments, ensuring reproducibility and robustness of the results.

## Results

Age-specific responses of hepatic expression of inflammatory sirtuins (*Sirt1* and *Sirt2*) after burn injury

**Figure 2:**
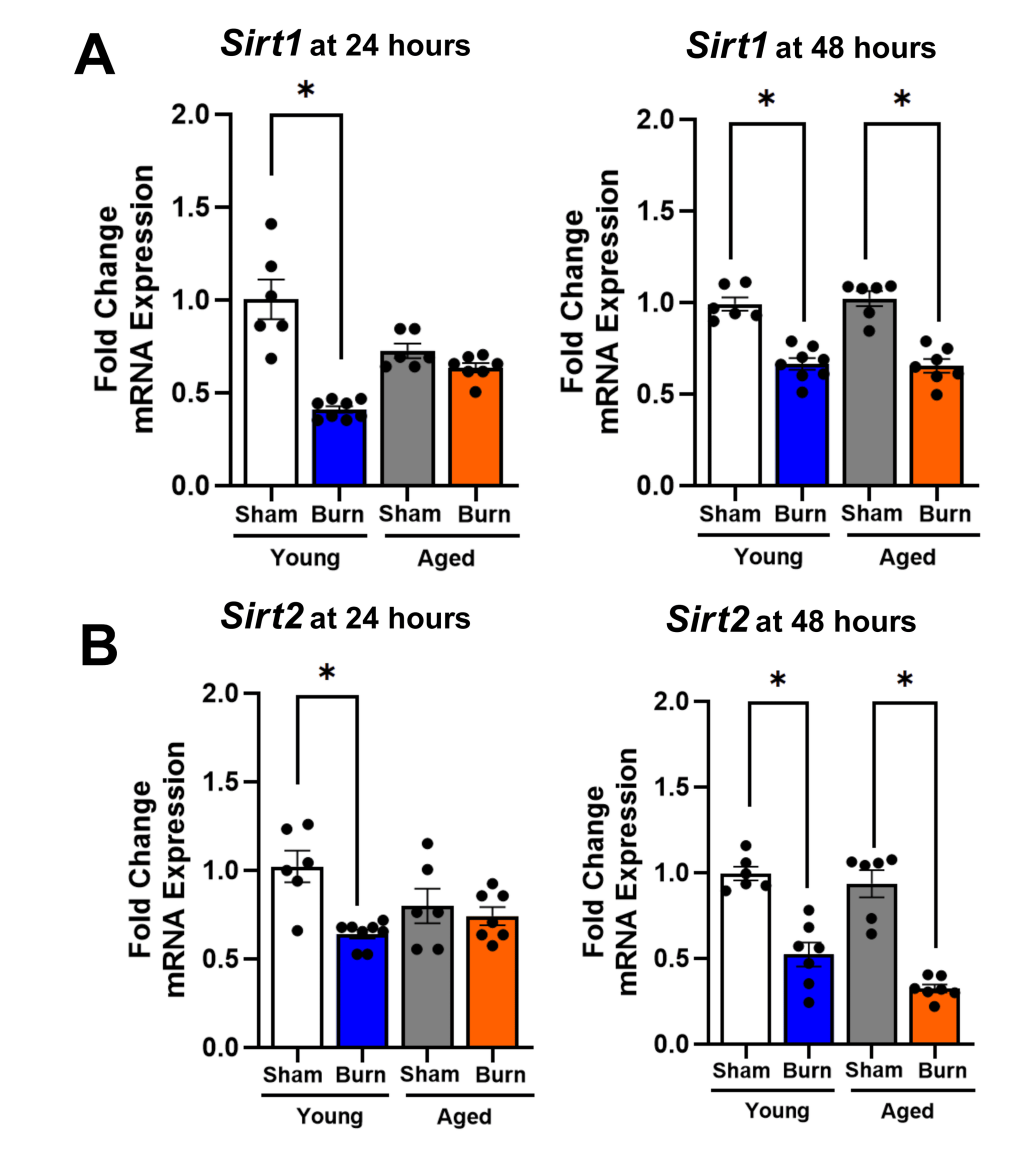
Inflammatory hepatic sirtuin (Sirt1-Sirt2) expression in young and aged mice after burn injury (A) *Sirt1* expression decreased at 24 hours (51%) and 48 hours (31%) in young burned mice, whereas aged burned mice showed no change at 24 hours and a reduction at 48 hours (34%). (B) *Sirt2 *declined at 24 hours (37%) and 48 hours (44%) in young burned mice, while aged burned mice exhibited no change at 24 hours, followed by a reduction at 48 hours (63%). Results were normalized using GAPDH as an internal control. Data are presented as mean ± SEM (n = 6-8 per group). Statistical significance was determined using one-way ANOVA followed by Tukey's post hoc test (*p < 0.05) compared to their respective sham controls. GAPDH: glyceraldehyde 3-phosphate dehydrogenase; SEM: standard error of the mean; ANOVA: analysis of variance

Quantitative RT-qPCR analysis revealed distinct temporal and age-dependent patterns in sirtuin expression following burn injury (Figure 2). *Sirt1* and *Sirt2*, which regulate inflammatory responses and nuclear factor-kappa B (NF-κB) signaling, showed notable differences between young and aged mice.

In young burn-injured mice, hepatic *Sirt1* expression was reduced by 51% at 24 hours post-burn when compared to livers from young sham-injured controls (p < 0.05). At 48 hours, young mice showed partial recovery, with *Sirt1* levels remaining 31% below sham-injured mice (p < 0.05). In contrast, at 24 hours after burn injury, the livers of aged mice failed to show significant changes in *Sirt1* expression at 24 hours relative to aged sham-injured mice and had a 34% reduction at 48 hours (p < 0.05). Two-way ANOVA revealed a significant interaction between age and burn injury (p = 0.023), confirming distinct age-dependent temporal responses.

Hepatic mitochondrial sirtuins (*Sirt3*, *Sirt4*, and *Sirt5*) showed delayed recovery in aged mice

**Figure 3:**
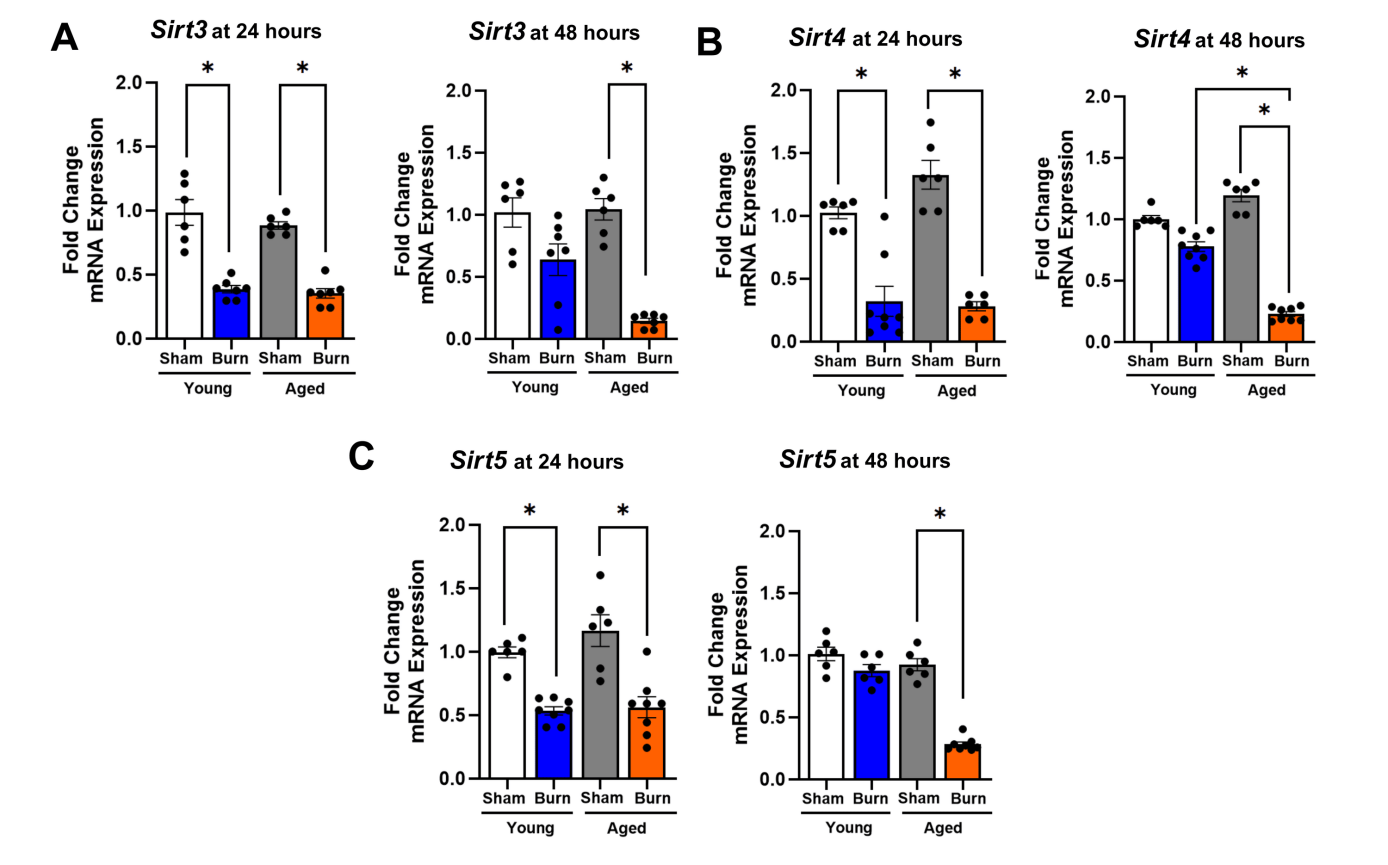
Mitochondrial hepatic sirtuin (Sirt3-Sirt5) expression in young and aged mice after burn injury (A) *Sirt3 *decreased at 24 hours in young (61%) and aged burned mice (59%), with suppression persisting at 48 hours only in aged mice (68%). (B) *Sirt4* expression dropped at 24 hours in young burned mice (64%) but returned to near-baseline at 48 hours, whereas aged burned mice exhibited a reduction at 24 hours (80%), persisting at 48 hours (76%). At 48 hours, *Sirt4* expression in young burned mice was higher than in aged burned mice (71%) (p < 0.05). (C) *Sirt5* expression decreased at 24 hours (23%) and remained unchanged at 48 hours in young burned mice, whereas aged burned mice showed a reduction at 24 hours (42%), which further declined at 48 hours (58%) (p < 0.05). Results were normalized using GAPDH as an internal control. Data are presented as mean ± SEM (n = 6-8 per group). Statistical significance was determined using one-way ANOVA followed by Tukey's post hoc test (p < 0.05) compared to their respective sham controls. *p < 0.05 for comparison between the young burn and aged burn groups. GAPDH: glyceraldehyde 3-phosphate dehydrogenase; SEM: standard error of the mean; ANOVA: analysis of variance

Mitochondrial sirtuins (*Sirt3*, *Sirt4*, and *Sirt5*) play essential roles in regulating oxidative stress and maintaining metabolic homeostasis. Our analysis revealed significant burn-induced alterations in their expression patterns with notable age-dependent differences in recovery kinetics (Figure 3).

Hepatic *Sirt3* expression was markedly suppressed at 24 hours after burn injury in both young (61% decrease) and aged (59% decrease) mice compared to their respective sham controls (p < 0.05). At 48 hours post-burn, young mice showed substantial recovery in liver *Sirt3* expression, returning to near-baseline levels (yet still 15% below young sham, p <0.05). In contrast, aged mice maintained persistent suppression with a 68% reduction compared to aged sham controls (p < 0.05). The difference in *Sirt3* expression between young and aged burned mice at 48 hours was statistically significant (p < 0.05).

*Sirt4* expression in the liver followed a similar pattern as seen with *Sirt3,* with decreases in both young (64%) and aged (80%) burned mice at 24 hours compared to their respective sham-injured controls (p < 0.05). At 48 hours after injury, young burned mice demonstrated remarkable recovery, with *Sirt4* levels returning to near-baseline (11% below sham levels, p > 0.05), whereas aged burned mice showed sustained suppression (76% below sham, p < 0.05). This resulted in* Sirt4* levels being 71% higher in young burned mice than aged burned mice at 48 hours (p < 0.05), representing the most pronounced age-dependent difference observed among all sirtuins. Hepatic expression of *Sirt5* decreased by 23% in young burned mice and 42% in aged burned mice at 24 hours after injury compared to their respective sham controls (p < 0.05). While young injured mice maintained this reduced level at 48 hours with no further decline (27% below sham, p < 0.05), aged injured mice exhibited progressive suppression with *Sirt5* levels decreasing by 58% at 48 hours post-burn compared to aged sham controls (p < 0.05). Direct comparison between age groups at 48 hours showed significantly lower *Sirt5* expression in aged burned mice (p < 0.05).

Genome stability sirtuins (*Sirt6* and *Sirt7*) show age-specific responses in the liver after burn injury

**Figure 4:**
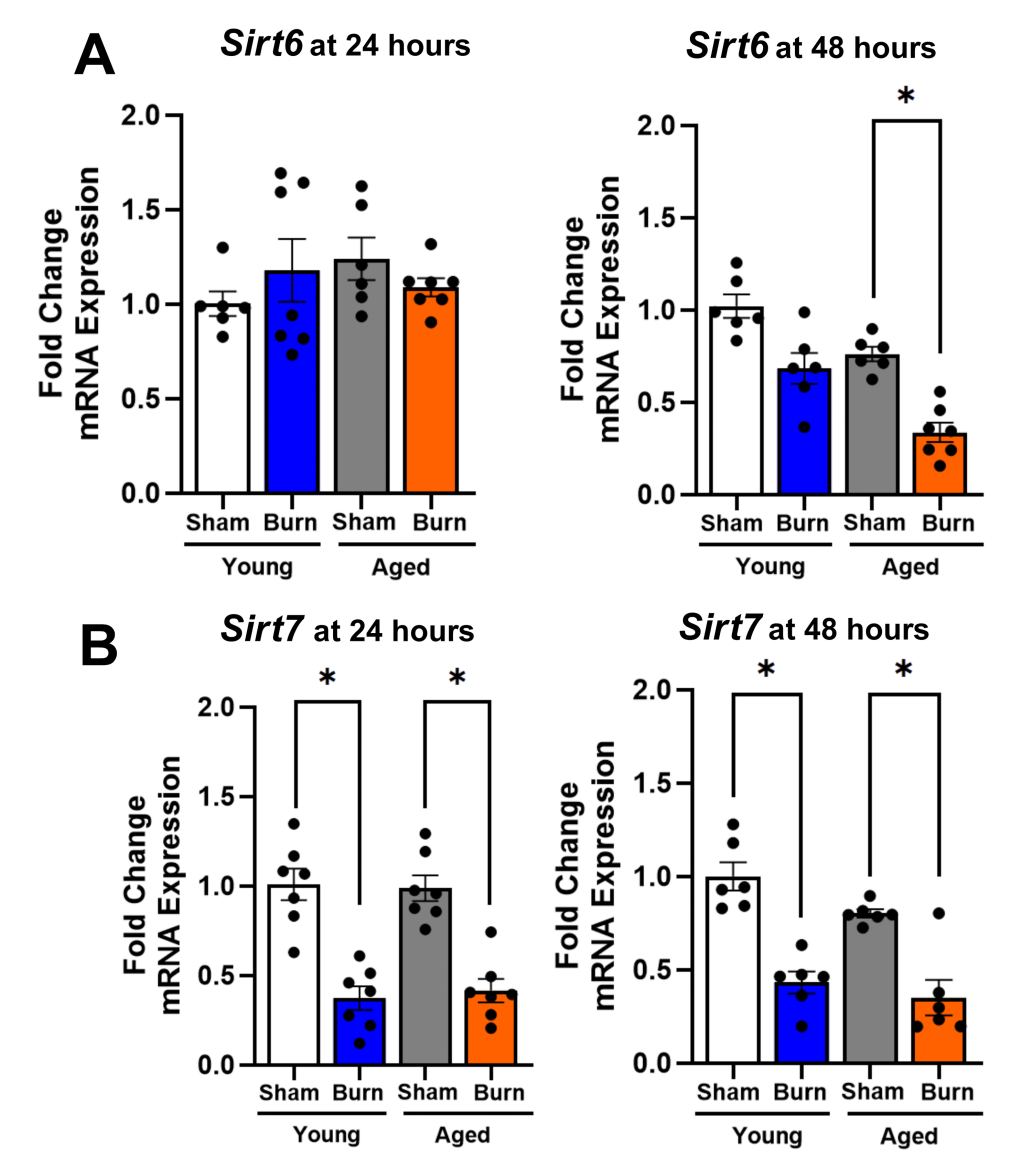
Hepatic genome stability sirtuin (Sirt6-Sirt7) expression in young and aged mice after burn injury (A) *Sirt6* remained unchanged at both points in young burned mice but showed a reduction at 48 hours in aged burned mice (45%). (B) *Sirt7* was significantly reduced at 24 (50%) and 48 hours (47%) in young burned mice and aged burned mice at 24 hours (55%) and 48 hours (54%). Results were normalized using GAPDH as an internal control. Data are presented as mean ± SEM (n = 6-8 per group). Statistical significance was determined using one-way ANOVA followed by Tukey's post hoc test (p < 0.05) compared to their respective sham controls. *p < 0.05 for comparison between the young burn and aged burn groups. GAPDH: glyceraldehyde 3-phosphate dehydrogenase; SEM: standard error of the mean; ANOVA: analysis of variance

*Sirt6* and *Sirt7,* which regulate genomic stability, DNA repair, and nuclear homeostasis, demonstrated distinct age-dependent responses to burn injury (Figure 4).

*Sirt6* expression in the liver remained unchanged in young mice at both 24 and 48 hours post-burn compared to young sham-injured controls (7% and 12% reductions, respectively, p > 0.05). On the other hand, this *Sirt* failed to change in the livers of aged burned mice at 24 hours post injury (15% reduction, p > 0.05). However, there was a substantial 45% reduction in *Sirt6* expression at 48 hours below that of aged sham controls (p < 0.05). Direct comparison between young and aged burned mice at 48 hours confirmed significantly lower *Sirt6* expression in aged animals (p < 0.05).

*Sirt7* expression was also reduced at both time points in the livers of young burn injured mice (24 hours: 50%, 48 hours: 47% reductions, respectively, p < 0.05) and aged burned mice (24 hours: 55%, 48 hours: 54% reductions, respectively, p < 0.05) compared to age-specific sham controls. While both age groups showed similar magnitudes of *Sirt7 *suppression, there was no evidence of recovery in either age group at 48 hours after injury, suggesting persistent disruption of nuclear homeostatic mechanisms following burn injury across all ages.

## Discussion

**Figure 5:**
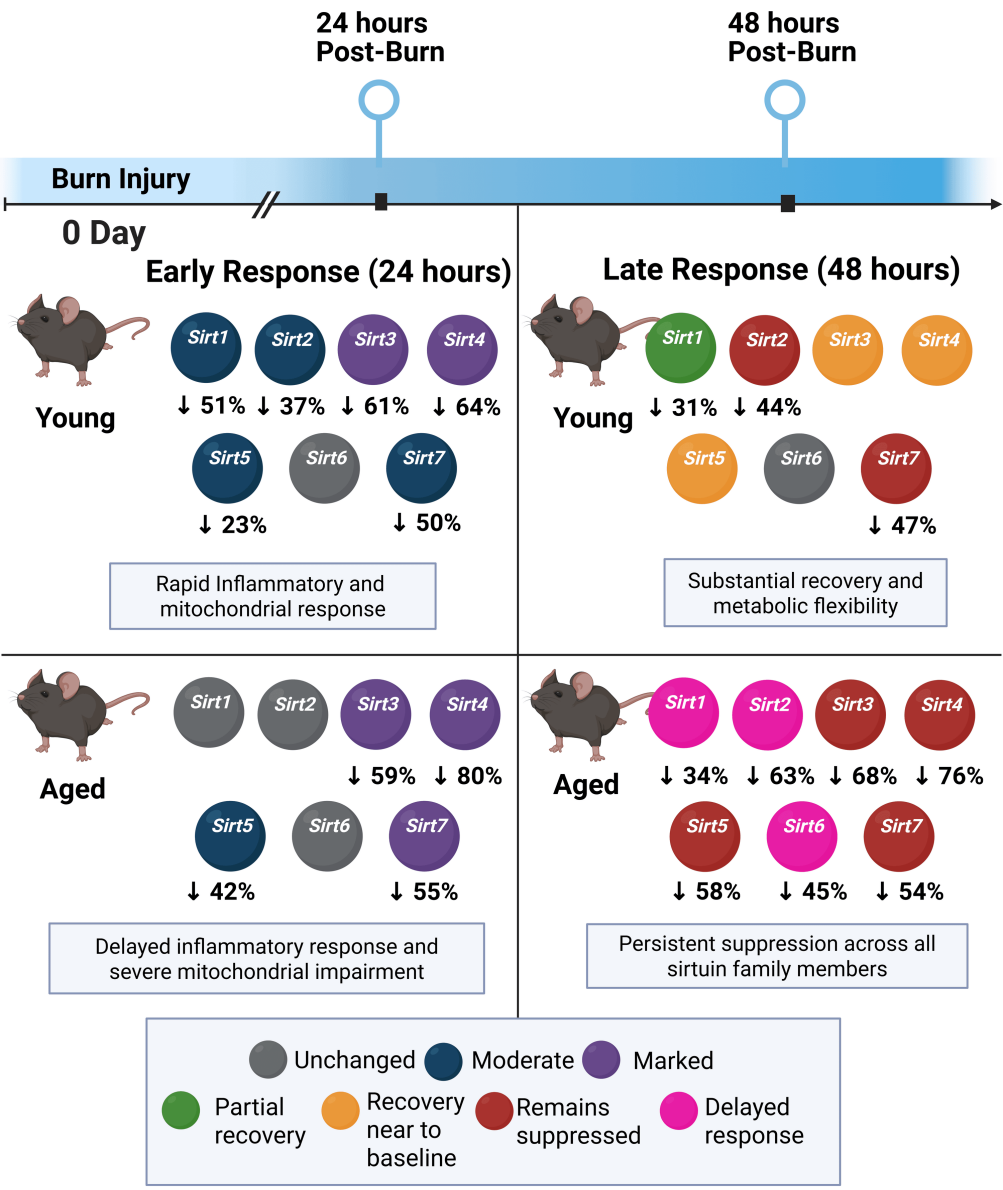
Integrated model of age-dependent hepatic sirtuin dysregulation and its impact on hepatic injury following burn trauma (A) Early response (24 hours): Young burn-injured mice show suppression of hepatic inflammatory and mitochondrial sirtuins at 24 hours after burn, with minimal impact on genomic stability sirtuins. Aged mice exhibit selective suppression of mitochondrial sirtuins with relative preservation of inflammatory regulators. (B) Late response (48 hours): Young burn-injured mice demonstrate substantial restoration of expression of *Sirt1*, *Sirt3*, and *Sirt4* in the liver, maintaining metabolic flexibility and inflammatory resolution. In contrast, aged burned mice show delayed and more profound and persistent suppression across all sirtuin families, leading to sustained inflammation, mitochondrial dysfunction, and impaired DNA repair. The pattern of expression of sirtuins in aged mice provides a possible molecular basis for the observed exacerbation of hepatic damage in elderly burn victims and identifies potential targets for therapeutic intervention. This image was created by the author (Juan-Pablo Idrovo) using BioRender (https://BioRender.com/y26b685).

Severe burn injuries trigger a systemic inflammatory response that affects multiple organs, including the liver, leading to significant morbidity and mortality [4,17]. Aging has been associated with increased susceptibility to hepatic injury, delayed recovery, and impaired metabolic adaptation following thermal trauma, highlighting a critical need for age-specific therapeutic strategies [5]. Our study demonstrates that burn injury significantly alters hepatic sirtuin expression in an age- and time-dependent manner, with aged mice exhibiting distinct patterns of sirtuin dysregulation compared to their younger counterparts. Given the essential role of sirtuins in regulating inflammation, oxidative stress, and metabolic homeostasis, these findings have important translational implications (Figure 5).

Inflammatory regulation: *Sirt1 *and *Sirt2*

**Figure 6:**
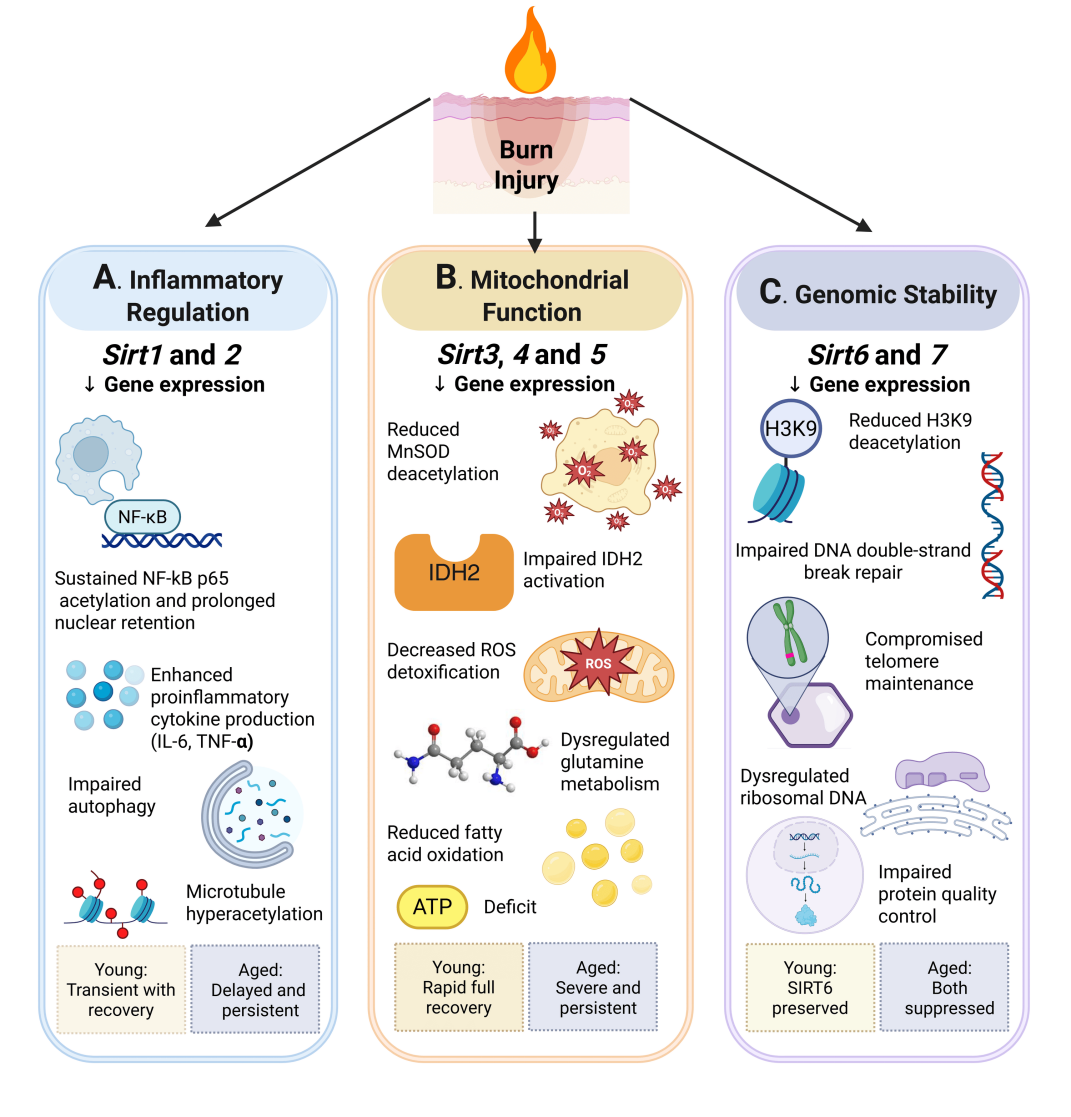
Schematic representation of the mechanistic implications of sirtuin dysregulation in age-associated burn response Age-dependent alterations in sirtuin expression following burn injury contribute to differential hepatic outcomes through distinct pathways: (A) Inflammatory regulation: Suppression of *Sirt1/2* leads to sustained NF-κB activation and amplified inflammatory responses [20-24]. (B) Mitochondrial function: Downregulation of *Sirt3/4/5* results in impaired oxidative stress defenses, metabolic inflexibility, and energy deficits [27-32]. (C) Genomic stability: Reduced *Sirt6/7* expression compromises DNA repair mechanisms and protein quality control [33-39]. In aged animals, the persistent suppression of these pathways leads to exacerbated and prolonged hepatic dysfunction following burn injury. NF-κB: nuclear factor-kappa B; IL-6: interleukin-6; TNF-α: tumor necrosis factor-alpha; MnSOD: manganese superoxide dismutase; IDH2: isocitrate dehydrogenase 2; ROS: reactive oxygen species; ATP: adenosine triphosphate This image was created by the author (Juan-Pablo Idrovo) using BioRender (https://BioRender.com/y26b685).

As mentioned above, *Sirt1* and *Sirt2* function as key negative regulators of inflammation through their inhibitory effects on NF-κB signaling, inflammasome activation, and pro-inflammatory cytokine production [20,21]. Our findings reveal that *Sirt1* and *Sirt2* expressions in the liver were significantly suppressed in aged burned mice at 48 hours, whereas young mice showed either partial recovery or less severe suppression (Figure 6A).

The functional consequences of attenuated *Sirt1* expression include sustained NF-κB p65 acetylation, which prolongs its nuclear retention and transcriptional activity, leading to excessive production of pro-inflammatory cytokines, such as interleukin-6 (IL-6) and tumor necrosis factor-alpha (TNF-α) [22]. Additionally, reduced *Sirt1* activity impairs autophagy-mediated clearance of damaged cellular components, further exacerbating inflammation and oxidative stress [23]. Similarly, *Sirt2* depletion promotes microtubule hyperacetylation and NF-κB activation, resulting in heightened inflammatory responses and macrophage infiltration [24]. The delayed and more profound suppression of these inflammatory regulatory sirtuins in the livers of aged mice suggests a mechanism for the exaggerated and prolonged inflammatory response observed in elderly burn victims.

Liver inflammation is a well-documented complication in burn patients, contributing to systemic metabolic dysregulation, immune dysfunction, and multi-organ failure [2,5,6,8,25]. The persistent suppression of *Sirt1* and *Sirt2* in the livers of aged burned mice suggests that aging weakens the ability of the liver to resolve inflammation, predisposing the host to sustain hepatic injury and metabolic imbalance post-burn. Clinically, reduced hepatic *Sirt1* expression has been linked to prolonged inflammatory signaling and impaired liver regeneration in critically ill and elderly patients [26], further supporting the translational relevance of our findings.

These observations align with our previous studies that demonstrated heightened inflammatory responses and elevated pro-inflammatory cytokine levels in the livers of aged mice following burn injury [5]. The correlation between sirtuin suppression and enhanced inflammatory responses provides new mechanistic insights into the age-dependent exacerbation of burn-induced liver damage.

Mitochondrial function: *Sirt3*, *Sirt4*, and *Sirt5*

Mitochondrial integrity is critical for energy metabolism and oxidative stress regulation, particularly following burn-induced cellular injury. *Sirt3*, *Sirt4*, and *Sirt5* play essential roles in mitochondrial adaptation by regulating specific aspects of mitochondrial function: *Sirt3* deacetylates and activates manganese superoxide dismutase (MnSOD) and isocitrate dehydrogenase 2 (IDH2), enhancing reactive oxygen species (ROS) detoxification; *Sirt4* regulates glutamine metabolism and mitochondrial adenosine triphosphate (ATP) production; and *Sirt5* desuccinylates and activates multiple metabolic enzymes involved in the urea cycle and oxidative defense [27]. Burn patients, especially elderly patients, exhibit prolonged mitochondrial dysfunction, increased oxidative stress, and impaired hepatic energy metabolism, which have been linked to worse outcomes and delayed recovery (Figure 6B) [28].

Data presented herein demonstrate that *Sirt3* suppression persisted in the livers of aged burned mice at 48 hours, while there is substantial recovery of expression in young burned mice. This prolonged reduction in *Sirt3* expression aligns with studies showing that the livers of aged individuals have reduced capacity to restore mitochondrial function efficiently, leading to increased oxidative stress and prolonged energy deficits [29]. Similar trends have been observed in burn patients, where decreased *Sirt3* expression correlates with impaired mitochondrial respiration and metabolic inflexibility, particularly in elderly individuals [30].

Likewise, *Sirt4* levels remained significantly suppressed in aged burned mice, whereas young burned mice exhibited remarkable recovery at 48 hours, suggesting that advanced age prolongs metabolic dysfunction and impairs adaptive responses. In burn patients, mitochondrial metabolic inflexibility has been associated with persistent catabolism and organ dysfunction, further highlighting the translational relevance of these findings [31]. *Sirt5*, which regulates ammonia detoxification and oxidative stress resistance through its desuccinylase activity, was also significantly reduced in aged burned mice, contributing to prolonged hepatic dysfunction. Similar impairments in nitrogen balance and oxidative stress defense mechanisms have been reported in critically ill burn patients, reinforcing the importance of mitochondrial sirtuins in hepatic resilience post-burn [32].

These findings complement our previous metabolomic analyses, which revealed significant disruptions in tricarboxylic acid cycle (TCA cycle) intermediates, energy metabolites, and redox homeostasis in aged mouse livers following burn injury [10]. The persistent suppression of mitochondrial sirtuins in aged mice provides a potential mechanism for the observed metabolic dysfunction and heightened oxidative stress in elderly burn victims.

Genomic stability and cellular stress: *Sirt6* and *Sirt7*

Genomic stability is essential for cellular survival and tissue recovery following burn injury, as extensive DNA damage and protein misfolding contribute to hepatic dysfunction and impaired regeneration. In burn patients, particularly the elderly, increased oxidative stress and epigenetic dysregulation have been linked to prolonged hepatic insufficiency and heightened susceptibility to organ failure (Figure 6C) [33].

*Sirt6 *and *Sirt7* play pivotal roles in maintaining genome integrity, chromatin remodeling, and protein quality control [34,35]. *Sirt6* deacetylates histone H3K9 to facilitate DNA double-strand break repair and regulate telomere maintenance, while *Sirt7* controls ribosomal DNA transcription and protein synthesis under stress conditions [36]. However, their expression was markedly suppressed in aged burned, and not young burned, mice at 48 hours post-injury. *Sirt6* depletion has been associated with reduced DNA repair efficiency, increased genomic instability, and accelerated aging of the liver through hyperactivation of NF-κB and impaired glycolytic regulation [37]. The data in this paper suggest that sustained *Sirt6* suppression in aged burned mice contributes to prolonged cellular stress and delays in hepatic regeneration. In burn patients, diminished *Sirt6* activity has been implicated in impaired wound healing, chronic inflammation, and fibrosis in burn patients, reinforcing its critical role in recovery [38].

Similarly, *Sirt7*, a key regulator of ribosomal biogenesis and proteostasis, remained significantly lower in aged burn-injured mice than in young injured animals, indicating a diminished capacity to restore protein homeostasis after trauma. In critically ill burn patients, prolonged endoplasmic reticulum (ER) stress and defective protein-folding mechanisms have been associated with persistent organ dysfunction and poor clinical outcomes [39].

These observations align with our previous histological findings of increased cellular damage and necrosis in the livers of aged mice following burn injury [10]. The limited expression of genome stability sirtuins provides a potential mechanism for the compromised cellular resilience and regenerative capacity observed in elderly burn patients.

Age-related impairment in sirtuin recovery and clinical implications

One of the key findings of this study is the marked difference in hepatic recovery between young and aged burned mice. Young mice exhibited transient sirtuin suppression followed by partial or complete recovery at 48 hours, whereas aged mice demonstrated delayed and prolonged downregulation, leading to liver damage. This pattern mirrors clinical observations in burn patients, where younger individuals exhibit a more robust metabolic response, better mitochondrial resilience, and faster resolution of inflammation, ultimately leading to improved hepatic recovery and overall survival [6].

These findings are consistent with previous research showing that younger humans have a greater capacity for metabolic adaptation, mitochondrial repair, and inflammatory resolution following burn injury [40]. In contrast, aged mice remain in a state of prolonged inflammation, mitochondrial dysfunction, and impaired DNA repair, leading to worsened hepatic outcomes. Similarly, clinical studies have reported that elderly burn patients experience prolonged hypermetabolism, increased oxidative stress, and greater susceptibility to liver dysfunction, contributing to higher rates of sepsis, organ failure, and mortality compared to comparably injured younger cohorts [41]. Additionally, older burn patients exhibit impaired hepatic protein synthesis, reduced antioxidant capacity, and persistent catabolic stress, all of which further exacerbate metabolic inefficiency and delay recovery [42].

The delayed recovery of sirtuins in aged burn-injured mice suggests that advanced age weakens the ability of the body to mount an effective stress response, increasing the risk of chronic liver dysfunction and multi-organ failure in elderly burn patients. Clinically, this is reflected in prolonged intensive care unit (ICU) stays, increased complications, and higher mortality rates among elderly burn victims, emphasizing the urgent need for targeted therapeutic strategies to enhance hepatic resilience in this vulnerable population [43].

Therapeutic implications and future directions

Expanding on the clinical significance of these findings, sirtuin modulation may present a promising therapeutic avenue for burn patients, particularly the elderly [44-46]. Pharmacological activation of sirtuins has shown potential in preclinical models for mitigating inflammation and oxidative stress. Resveratrol, a well-studied *Sirt1* activator, has demonstrated protective effects against burn-induced inflammation and organ damage in experimental models [47]. Similarly, NAD^+^ precursors, such as nicotinamide riboside and nicotinamide mononucleotide, can enhance sirtuin activity by increasing cellular NAD^+^ levels, potentially improving mitochondrial function and reducing oxidative stress in aged tissues [11].

Based on our findings, targeting specific sirtuin pathways could help preserve mitochondrial function, enhance metabolic adaptation, and reduce prolonged inflammatory states post-burn. For example, *Sirt3* activators might be particularly beneficial in preserving mitochondrial integrity and reducing oxidative stress in elderly burn patients. Similarly, *Sirt1* and *Sirt2* agonists could help attenuate excessive inflammation and promote resolution in the aged liver. Although preclinical studies have shown encouraging results, no clinical trials are currently underway investigating sirtuin modulators in human burn patients. Thus, further research is warranted to explore the safety, efficacy, and translational potential of these compounds, with the ultimate goal of integrating sirtuin-based therapies into clinical protocols to improve hepatic resilience and outcomes in this vulnerable population.

Additionally, identifying clinical biomarkers for the activity of sirtuins in patients with severe burns could facilitate early intervention and individualized treatment strategies. Measuring circulating levels of sirtuin-regulated metabolites or plasma markers of mitochondrial dysfunction might help identify patients at highest risk for age-associated complications such as persistent hepatic inflammation, metabolic dysregulation, multi-organ failure, and delayed wound healing. By detecting these risks early, clinicians could implement targeted therapies to mitigate organ dysfunction, improve recovery trajectories, and ultimately reduce morbidity in elderly burn patients.

Limitations and future research

Several limitations of this study should be acknowledged to guide future research. First, our study was conducted exclusively in female mice due to the established sex-based differences in burn responses and inflammatory regulation [42]. While this approach enhances internal consistency, it limits generalizability to male subjects. Future studies should compare sirtuin responses in both sexes to develop sex-specific therapeutic approaches.

Second, while the 15% TBSA murine burn model used is well-established and clinically relevant [3,12,13,18], it does not fully replicate the multisystem trauma and physiological heterogeneity observed in human burn patients. Expanding research to include combined injury models, such as burn with infection or ischemia-reperfusion injury, could enhance the clinical relevance of these findings.

Third, our analysis focused on sirtuin gene expression, which provides valuable insights into transcriptional regulation and does not necessarily reflect protein abundance or enzymatic activity. Future studies should incorporate proteomic analysis and enzymatic activity assays to confirm that transcriptional changes translate to functional alterations in sirtuin activity.

Fourth, the 24- and 48-hour timepoints provide critical information about the acute phase response. However, they limit our ability to assess long-term hepatic adaptations, including the development of fibrosis, sustained metabolic dysfunction, and regenerative capacity in aged mice. Extended time points would be valuable for evaluating whether persistent sirtuin dysregulation contributes to chronic hepatic impairment post-burn.

Finally, while we have identified important correlations between sirtuin expression patterns and age-dependent hepatic vulnerability, the present study does not establish a causative relationship due to its observational design and reliance on gene expression data without functional validation. Establishing causality requires mechanistic studies that go beyond association. Future investigations involving genetic knockout models or pharmacological modulation of specific sirtuins in aged mice will be critical to determine whether restoring or enhancing sirtuin activity can directly mitigate burn-induced liver damage and improve clinical outcomes.

## Conclusions

Our study demonstrates that burn injury induces significant alterations in hepatic sirtuin expression, with distinct age and time-dependent patterns. Aged mice exhibit delayed and more persistent suppression of hepatic sirtuin expression compared to their young counterparts, particularly affecting mitochondrial and genome stability sirtuins. These molecular changes provide a potential mechanistic explanation for the exacerbated liver damage, heightened oxidative stress, and impaired metabolic function previously observed in aged burn animals. The dysregulation of inflammatory sirtuins (*Sirt1* and *Sirt2*) may contribute to prolonged systemic inflammation, while suppressing mitochondrial sirtuins (*Sirt3-5*) likely impairs energy metabolism and oxidative stress resistance. The selective downregulation of *Sirt6* in aged mice following burn injury suggests compromised DNA repair and cellular resilience mechanisms that may further exacerbate tissue damage.

These findings establish a molecular basis for understanding age-associated hepatic vulnerability following burn injury and identify sirtuins as potential therapeutic targets. By developing interventions that enhance sirtuin activity or compensate for their diminished function, we may be able to improve hepatic resilience, attenuate organ dysfunction, and ultimately enhance clinical outcomes in elderly burn patients. Future translational research could focus on validating these findings in human subjects and developing age-appropriate therapeutic strategies to reduce the disproportionate burden of burn-related morbidity and mortality in the elderly population.

## Figures and Tables

**Figure 7:**
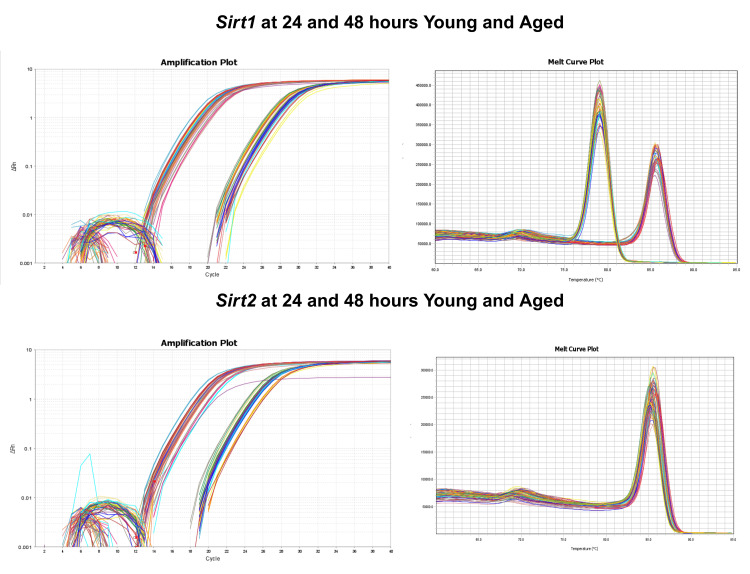
Amplification and melt curve analyses for Sirt1 and Sirt2 qPCR reactions qPCR: quantitative polymerase chain reaction

**Figure 8:**
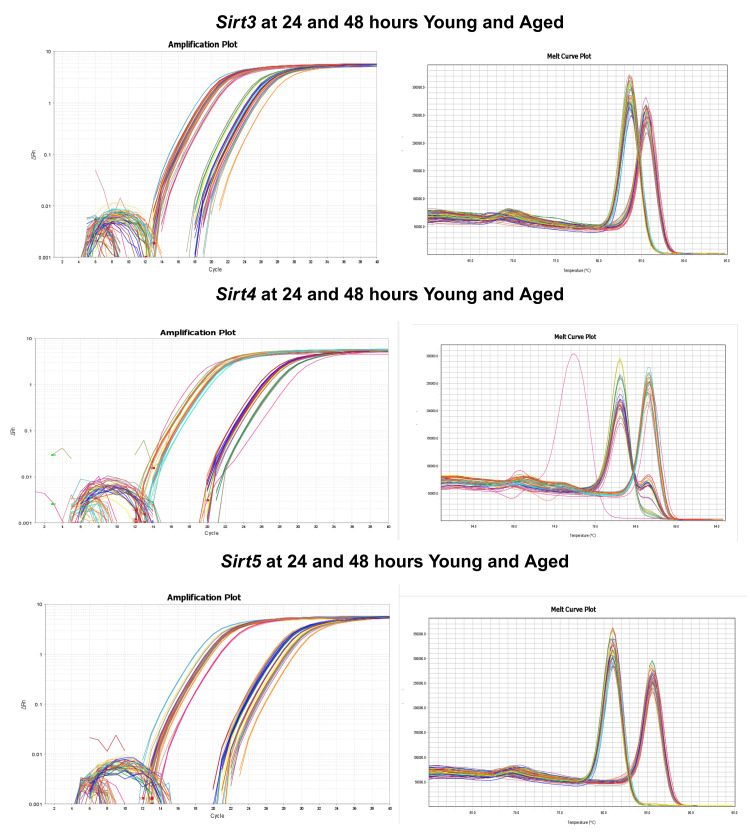
Amplification and melt curve analyses for Sirt3, Sirt4, Sirt5 qPCR reactions qPCR: quantitative polymerase chain reaction

**Figure 9:**
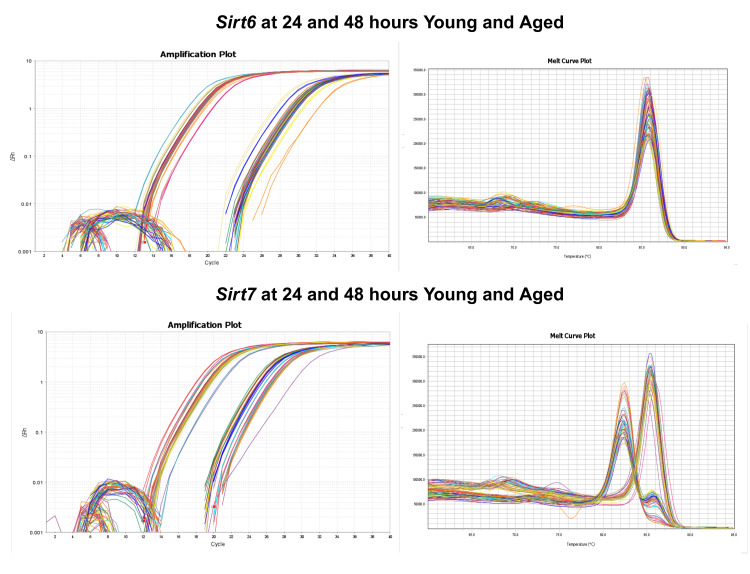
Amplification and melt curve analyses for Sirt6 and Sirt7 qPCR reactions qPCR: quantitative polymerase chain reaction

## Appendices

Appendix A 

Appendix B 

Appendix C 
